# Emergency MRI in acute dizziness and headache: current insights and future directions

**DOI:** 10.3389/fneur.2026.1906956

**Published:** 2026-08-10

**Authors:** Tatu Happonen, Mikko Nyman, Pauli Ylikotila, Jussi Hirvonen

**Affiliations:** 1 Department of Radiology, Turku University Hospital and University of Turku, Turku, Finland; 2 Neurocenter, Turku University Hospital and University of Turku, Turku, Finland; 3 Department of Radiology, Tampere University, Tampere, Finland

**Keywords:** dizziness, emergency imaging, headache, magnetic resonance imaging, neuroimaging

## Abstract

Acute dizziness and headache are common yet diagnostically complex symptoms in emergency departments, with causes ranging from benign to life-threatening. MRI reveals a broad spectrum of pathologies, though accessibility constraints and challenges in patient selection currently limit its use in emergency settings. This narrative review explores the role of emergency MRI in acute dizziness and headache, discusses its diagnostic impact and outlines priorities for future research.

## Highlights

- In acute dizziness, available evidence suggests that MRI is more accurate and may be more cost-effective than CT.
- In acute headache, existing data remain insufficient to reliably guide imaging decisions.
- Future challenges lie in limited accessibility, high costs, and complexities in patient selection.

## Introduction

### Clinical aspects of dizziness

In the International Classification of Vestibular Disorders, dizziness and vertigo are defined as distinct entities: dizziness refers to a sensation of impaired spatial orientation, while vertigo describes a sensation of distorted self-motion (1). In clinical practice, however, these terms are often used interchangeably due to overlapping symptom descriptions. For the sake of clarity and consistency, this review uses “dizziness” as an umbrella term encompassing sensations such as vertigo, unsteadiness, lightheadedness, and imbalance.

Dizziness is a rather common complaint, accounting for 2–3% of all emergency department (ED) visits (2–4). Patients exhibiting acute dizziness often represent a diagnostic challenge, as the differential diagnosis is broad. Although many patients are diagnosed with benign peripheral vestibular disorders, some require neuroimaging to assess for potential central causes. Stroke, particularly vertebrobasilar acute ischemic stroke, is the primary differential diagnosis among central causes, and is diagnosed in approximately 3–5% of ED visits for dizziness (5, 6). Despite the use of bedside examination patterns such as HINTS (Head Impulse, Nystagmus, Test of Skew) and STANDING, and clinical risk scores such as TriAGe+ and ABCD^2^, approximately 30–50% of acutely dizzy emergency patients undergo neuroimaging (6–9). This suggests that clinical tools alone may not always provide sufficient confidence to exclude the need for imaging, contributing to frequent imaging use.

A traditional paradigm to dizziness has relied on defining the type of perceived symptom (e.g., dizziness, vertigo, disequilibrium, presyncope, lightheadedness). This approach has shortcomings, as patients often have difficulty distinguishing between different types of dizziness and commonly alter their description when asked even minutes later, and that many simultaneously endorse multiple symptom descriptors (10, 11). Accumulating evidence suggests an approach based on symptom timing and triggers to guide imaging decisions and patient management (12–14).

### Clinical aspects of headache

Headache disorders rank among the most prevalent and disabling conditions worldwide, with an estimated global prevalence of 52% (15). Similar to dizziness, non-traumatic headache is a common complaint in the EDs, reported in 1–4% of all ED visits (16, 17). While most presentations of headache are benign and resolve spontaneously or with minor therapeutic measures, it is crucial to consider the possibility of potentially life-threatening secondary causes that may require further diagnostic workup. The differential diagnosis of secondary headache is vast, and involves conditions such as intracranial hemorrhages, cerebral or cerebellar infarctions, cerebral venous sinus thrombosis (CVST), arterial dissections, (non)ruptured aneurysms, infectious conditions, and brain tumors (18).

When history, physical, or neurologic examination elicit “red flags,” further investigation with imaging may be warranted to exclude a secondary cause. Red flags are widely described in the literature and include neurologic deficits, decreased consciousness, history of neoplasm, sudden or abrupt onset, association with activity or position, and older age (onset >65 years, or >50 years in some sources), among others (19, 20). However, the sensitivities, and especially the specificities, of these clinical features remain unclear due to the lack of prospective epidemiological studies.

### Emergency neuroimaging

Computed tomography (CT) is currently the most commonly used method in emergency neuroimaging, primarily due to its fast scan times, widespread availability, and lower cost compared to magnetic resonance imaging (MRI). CT is often the first-line imaging modality due to its high accuracy in detecting intracranial hemorrhage and its ability to suggest other acute pathologies that influence patient treatment in the emergency setting. However, CT does have several limitations. In stroke diagnostics, CT performs poorly especially in the posterior cranial fossa, with a low sensitivity of acute ischemic stroke (AIS) of ~30% in acute dizziness (21). Missing an acute stroke may result in an increased risk of future, potentially more severe infarcts. Furthermore, uncertainty in ruling out AIS with CT may result in stroke-mimicking conditions, such as migraine or vestibular disorders, being initially treated as a stroke. This may lead to unnecessary thrombolysis, which carries an inherent risk of intracranial hemorrhage, although the risk appears to be significantly lower in stroke mimics than in patients with true ischemic stroke, and may also increase costs and resource utilization (22). In the brain parenchyma, CT has limited sensitivity to detect subtle changes, such as those seen in very recent or lacunar strokes, infections, and demyelinating diseases. Despite the low sensitivity of CT for AIS in acute dizziness, combining CT, CT angiography, and CT perfusion might increase the sensitivity of diagnosing acute posterior circulation stroke to as high as 76% (23, 24). However, CT perfusion has important limitations in acutely dizzy patients, including limited sensitivity for small infarcts, particularly in the posterior fossa, as well as technical challenges related to posterior fossa imaging, such as beam-hardening artifacts and limited anatomical coverage. Early identification of acute stroke with these imaging modalities may facilitate timely treatment decisions, including intravenous thrombolysis and endovascular thrombectomy, in eligible patients.

Compared to CT, MRI offers superior soft tissue contrast and does not expose patients to ionizing radiation. In emergency neuroimaging, its higher sensitivity to subtle brain pathologies often leads to more accurate and earlier diagnoses. MRI is particularly feasible for the evaluation of vascular pathology, infection, cerebrospinal fluid abnormalities, demyelinating disease, and degenerative disorders. MRI is capable of detecting small infarcts, and it provides superior visualization of structures within the posterior fossa compared to CT. However, MRI’s use in emergency settings faces challenges due to limited accessibility, higher cost and longer scan times. There are also important limitations related to the early use of MRI, particularly diffusion-weighted imaging (DWI)-MRI in acutely dizzy patients. Small posterior circulation infarcts are particularly prone to being missed on early DWI-MRI, as they are substantially more likely to be DWI-negative than anterior circulation infarcts (25). Consequently, up to 20% of vertebrobasilar strokes may be missed on early DWI-MRI obtained within the first 24–48 h after symptom onset (21). The sensitivity of DWI-MRI is time dependent, emphasizing the importance of repeat MRI in patients with persistent clinical suspicion despite an initially negative MRI. Furthermore, a dedicated neuro-otological bedside examination, including HINTS when appropriately applied by trained clinicians, may demonstrate higher sensitivity for AIS than early MRI in selected patients (26). Optimized MRI protocols, including thin-section DWI, may further improve diagnostic performance for posterior circulation stroke (27).

Given the frequent neuroimaging use in acute dizziness and headache, understanding in which situations MRI offers the most diagnostic value is critical to balancing imaging yield with resource efficiency. This review examines current evidence of which patients with acute dizziness or headache are most likely to present with clinically significant MRI findings, and outlines directions for future research.

## Emergency MRI in dizziness

**Figure 1:**
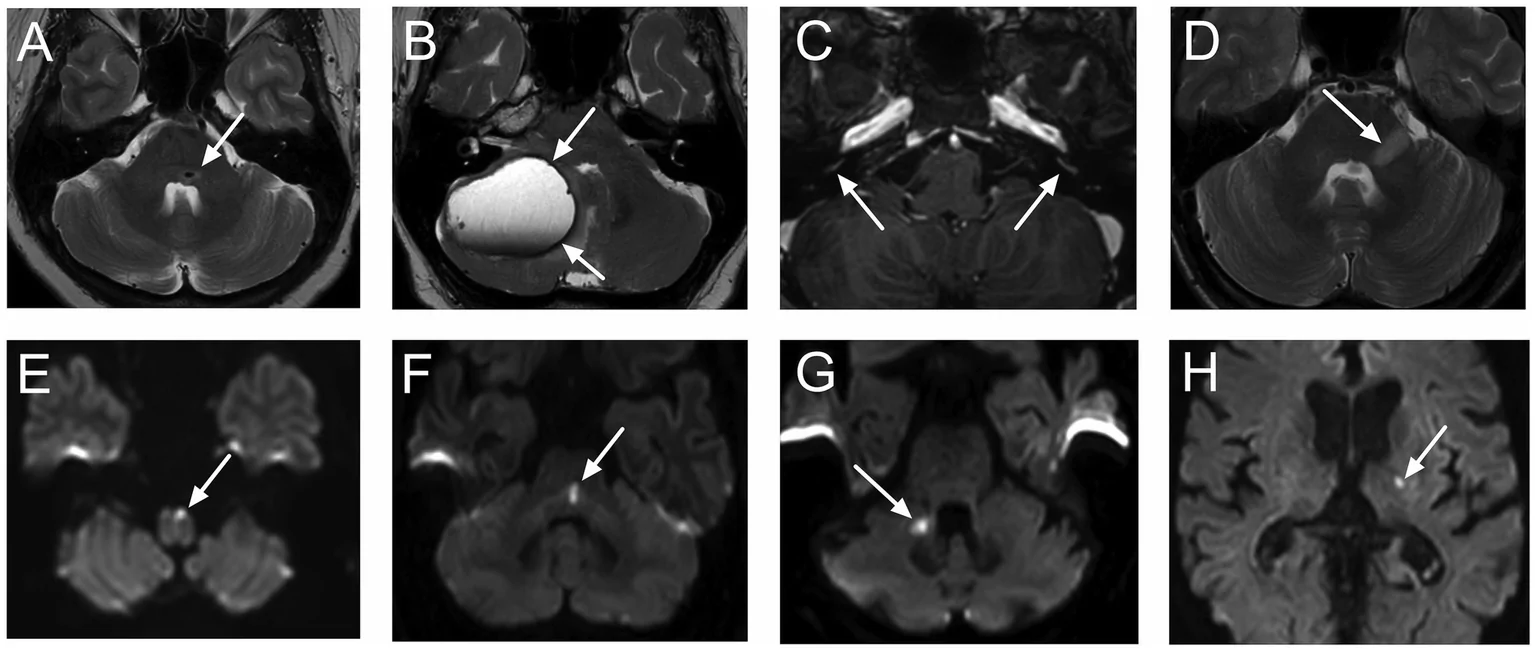
Representative emergency MRI findings in patients presenting to the emergency department with acute dizziness, illustrating the spectrum of clinically significant central pathologies detectable with MRI. Top row, non-ischemic findings: Pontine intracerebral hemorrhage (**A**, T2-weighted image; 55-year-old woman); cerebellar metastasis from lung cancer (**B**, T2-weighted image; 70-year-old woman); bilateral cochlear enhancement consistent with labyrinthitis (**C**, contrast-enhanced T1-weighted image; 45-year-old man); and an acute multiple sclerosis demyelinating lesion at the junction of the pons and the middle cerebellar peduncle (**D**, T2-weighted image; 24-year-old man); bottom row, acute ischemic infarcts on trace diffusion-weighted imaging: medulla oblongata (**E**, 67-year-old man), pons (**F**, 57-year-old man), cerebellum (**G**, 84-year-old man), and thalamus (**H**, 88-year-old man). White arrows indicate the relevant findings.

According to a recent study based on a large cohort of over 1,100 patients, MRI reveals a wide range of pathologies among acutely dizzy patients (28) (Figure 1). Approximately one in six scanned patients (17%) had an acute stroke. Interestingly, acute infarcts were also seen in patients whose only presenting symptom was dizziness. Among the ~200 patients with AIS on MRI, a preceding head CT scan suggested acute pathology only in 36% of the patients, highlighting the low yield that CT has among dizzy patients.

In the same study, significant non-ischemic findings included tumors and infections among other rare conditions, such as neurosarcoid, and were found in 8% of patients. Predictors of any acute pathology on MRI were older age (generally >55 years), male sex, cardiovascular risk factors and neurological signs. Similar risk factors have been reported by Kabra et al. and Machner et al., and are now further established in a larger patient cohort (29, 30). An important notion also highlighted by Machner and colleagues is that cardiovascular risk factors (such as older age, hypertension, and diabetes) do not seem as reliable in predicting AIS than neurological signs and therefore should not solely trigger imaging studies. Instead, proper history taking and careful clinical examination including assessment of neurological signs should guide the diagnostic pathway. Accumulating evidence suggests classifying patients into dizziness syndromes based on symptom timing and triggers, each with distinct differential diagnosis and imaging strategies (12, 14, 31).

Ideally, MRI would be preferred as the first-line imaging modality, considering the lower yield of CT among dizzy patients. Yet, most hospitals have limited access to emergency MRI, and therefore rely more heavily on CT. Indeed, if a pretest probability of stroke at 25% was applied on patients with acute vestibular syndrome (a clinical dizziness syndrome), a negative CT would only decrease the posttest probability to 19.4% (12). In addition, recent evidence endorses MRI as a more cost-effective method compared to CT or CT angiography for emergency neuroimaging of dizziness (32, 33). Taken together, MRI is likely more accurate and may be more cost-effective than CT for acutely dizzy patients who require neuroimaging.

## Emergency MRI in headache

**Figure 2:**
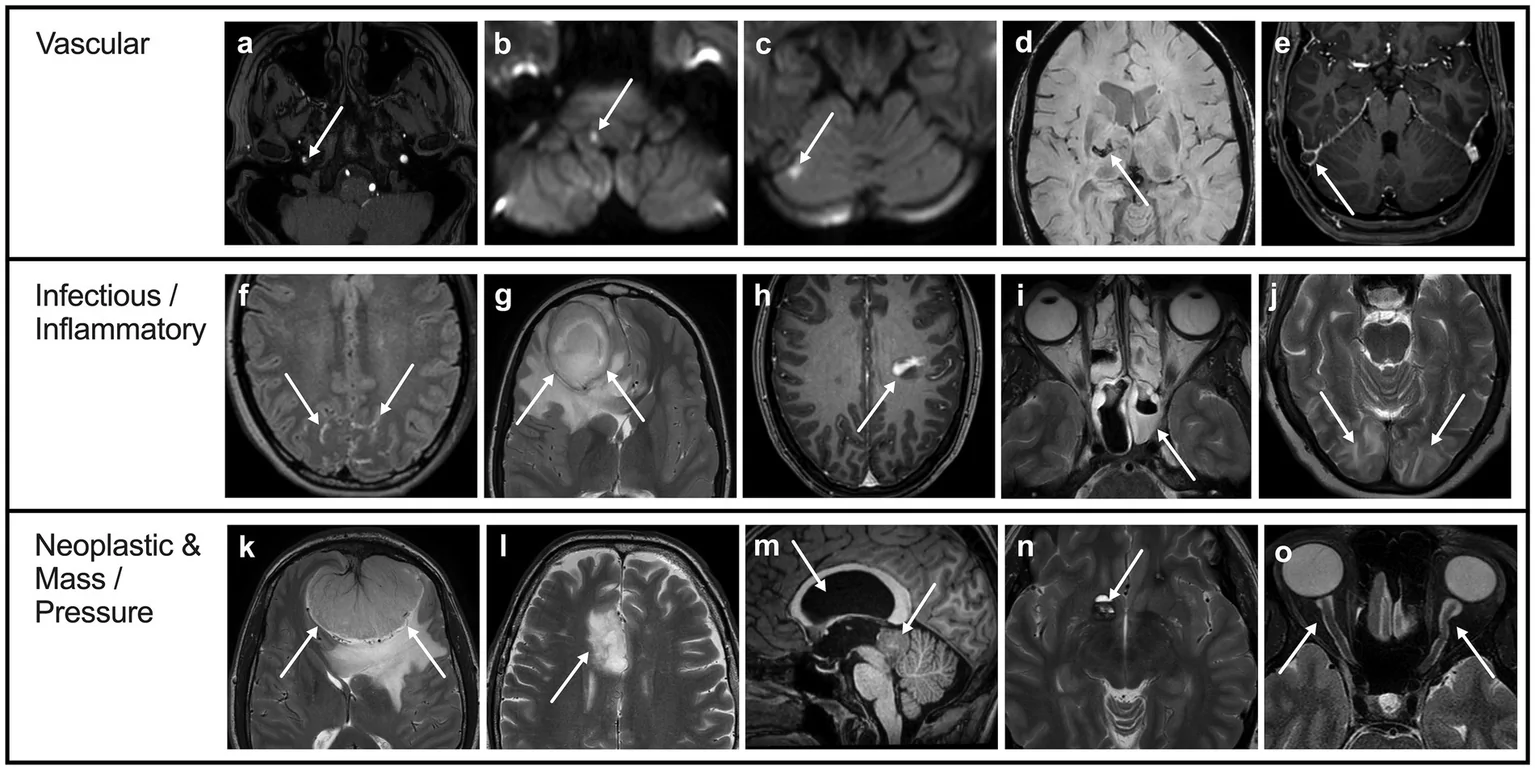
Emergency MRI findings considered significantly related to headache, grouped by etiology. Vascular causes (top row): internal carotid artery dissection **(a)**, small infarcts **(b,c)**, intracerebral hemorrhage **(d)**, and dural venous sinus thrombosis **(e)**. Infectious and inflammatory causes (middle row): Meningitis **(f)**, abscess **(g)**, demyelinating lesion **(h)**, sphenoid sinusitis **(i)**, and posterior reversible encephalopathy syndrome **(j)**. Neoplastic and mass- or pressure-related causes (bottom row): Meningioma **(k)**, glioma **(l)**, central neurocytoma with hydrocephalus **(m)**, cavernoma **(n)**, and idiopathic intracranial hypertension **(o)**. White arrows denote relevant findings. Adapted from Happonen et al. (34).

A retrospective study of approximately 700 patients demonstrated that MRI can reveal a wide range of intracranial pathologies in ED patients presenting with headache, although the majority of those scanned do not have clinically significant findings (34). The most frequent significant findings included infarcts, hemorrhages, tumors, sinusitis, and intracranial hypertension (Figure 2). In that study, the rate of detecting clinically significant pathology was 20% among all scanned ED patients with headache. For comparison, detection rates have been reported as lower, under 10%, in patients suspected of having a primary headache (i.e., a benign cause such as tension-type headache or migraine) (35, 36). Importantly, for patients presenting with worrisome symptoms, a normal MRI may provide reassurance and support safe discharge from the ED, underscoring the value of MRI not only in detecting pathology but also in excluding serious causes of headache.

Risk factors associated with clinically significant MRI findings in ED patients with headache include age over 40 years, smoking, signs or symptoms of infection, and nausea (34). Notably, patients with a history of migraine were less likely to have significant findings, suggesting they may undergo imaging more frequently despite a lower likelihood of serious pathology.

Incidental MRI findings are common in headache patients, occurring in 20–25% of scans (34, 37). While often benign, these findings may lead to unnecessary concern for patients and healthcare providers.

Published evidence on the early use of MRI in ED patients with headache remains limited. Outside the ED setting, a retrospective study of 82 walk-in clinic patients with acute headache, but low suspicion for intracranial bleeding, identified syncope, vomiting, ophthalmologic symptoms, and female sex as risk factors for significant MRI findings (36). While these studies offer valuable insights, the current evidence remains insufficient to reliably guide imaging decisions in headache patients, highlighting the need for further prospective research.

In patients imaged for suspected CVST, diagnostic yield remains modest despite frequent use of MRI. A retrospective study found signs of CVST in only 1.5% of cases, and clinically significant pathology overall in approximately 12%, with most scans remaining unremarkable (38). Compared to an earlier study using CT, the higher sensitivity of MRI did not translate into a considerably greater yield of significant findings (39). Although MRI offers higher accuracy in detecting and excluding acute pathology, CT often suffices in the emergency setting by identifying clinically important abnormalities with sufficient sensitivity, making both modalities viable for imaging suspected CVST.

## Discussion

### Clinical value of emergency MRI

MRI is particularly valuable in areas where CT has limitations. This is especially relevant in suspected stroke among patients presenting with acute dizziness, as CT has a sensitivity of only ~30% for AIS (21). Accurate and timely diagnosis is critical, as missed strokes may result in recurrent infarction or life-threatening complications such as brainstem compression.

In acute headache, the clinical value of emergency MRI is less clearly established. Although a wide range of clinically significant pathologies are detectable with MRI, current evidence does not identify a distinct subgroup of headache patients with consistently pronounced benefit from emergency MRI, as is the case for suspected stroke in acute dizziness. Importantly, in patients presenting with concerning symptoms, a normal MRI may provide reassurance and support safe discharge from the ED, underscoring its value not only in detecting but also in excluding serious intracranial causes of headache.

### Limitations of current evidence

The current evidence has several limitations. Most studies evaluating emergency MRI in acute dizziness and headache are retrospective, originate from single centers, and include only patients referred for emergency MRI, potentially introducing selection bias and limiting the generalizability of findings to patients who were not selected for imaging. Moreover, much of the literature focuses on diagnostic yield rather than outcomes such as changes in patient management, healthcare resource utilization, or cost-effectiveness. Prospective and ideally multicenter studies are needed to better establish the clinical and economic impact of emergency MRI.

### Future directions

**Figure 3:**
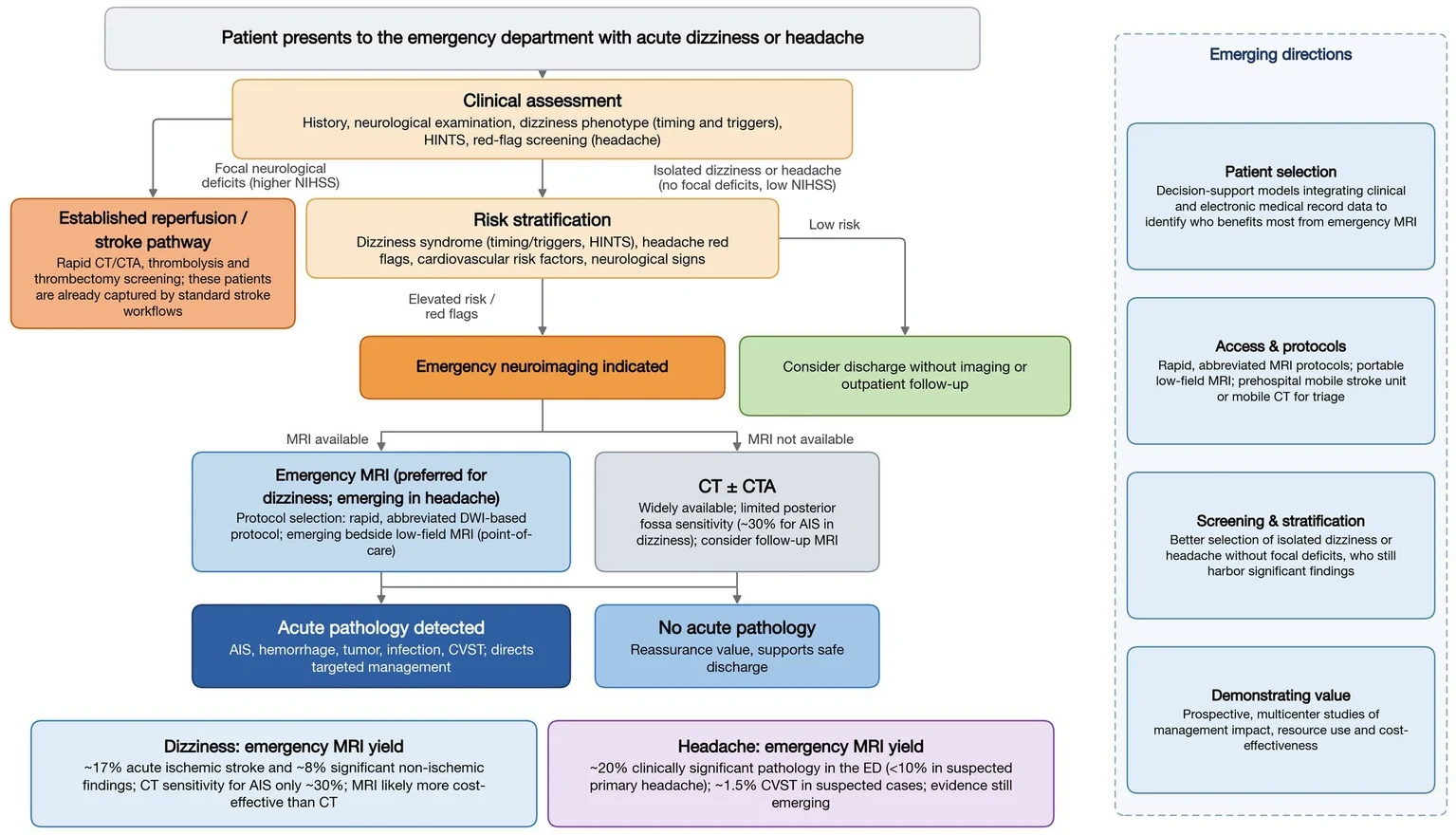
Conceptual workflow for emergency neuroimaging in acute dizziness and headache, showing the current pathway and emerging directions. AIS, acute ischemic stroke; CT, computed tomography; CTA, CT angiography; CVST, cerebral venous sinus thrombosis; DWI, diffusion-weighted imaging; HINTS, Head Impulse, Nystagmus, Test of Skew; MRI, magnetic resonance imaging; NIHSS, National Institutes of Health Stroke Scale.

Future advances in emergency MRI will likely depend on improving accessibility, refining patient selection, and demonstrating clinical value beyond diagnostic yield (Figure 3). Rapid MRI protocols, advances in image acquisition and reconstruction, and emerging portable low-field MRI systems may improve the practicality of MRI in emergency settings and expand access to advanced neuroimaging. These technologies may serve distinct implementation models. In the prehospital setting, mobile CT deployed in ambulances or mobile stroke units can accelerate triage of suspected stroke, although its value in isolated dizziness or headache without focal neurological deficits is likely limited. Within the emergency department or intensive care unit, bedside low-field MRI may instead enable point-of-care assessment and triage, potentially extending MRI access to patients for whom transfer to a conventional scanner is impractical.

At the same time, better methods are needed to identify patients most likely to benefit from emergency MRI. Current clinical tools provide only limited guidance, and future decision-support models incorporating clinical presentation and electronic medical record data may help optimize patient selection. Prospective studies should also evaluate whether improved diagnostic performance translates into meaningful benefit in patient management and healthcare resource utilization.

Future studies should distinguish between patients presenting with isolated dizziness or headache and those with accompanying focal neurological deficits. While patients with focal neurological deficits are often rapidly incorporated into established stroke imaging pathways, patients with isolated symptoms may enter diagnostic and treatment pathways with a delay, despite a meaningful proportion harboring clinically significant findings, including acute ischemic stroke. Further research may help optimize the imaging pathways and patient selection for emergency MRI in patients presenting without focal neurological deficits.

## Conclusion

Emergency MRI is a valuable diagnostic tool in the evaluation of acute dizziness and headache, though its use in emergency settings remains primarily limited by accessibility constraints. In acute dizziness, available evidence suggests that MRI is more accurate and may be more cost-effective than CT. While the diagnostic role of emergency MRI in acute headache is still emerging, current evidence remains insufficient to reliably guide imaging decisions in this patient group. Future efforts should focus not only on improving access to MRI, but also on identifying the patients most likely to benefit from emergency imaging. In cases where imaging is warranted, MRI offers high diagnostic accuracy for both detecting and ruling out serious intracranial pathology.
